# The Role of Renal Na^+^/H^+^ Exchange in the Regulation of Acid-Base and Sodium Homeostasis in Premature Infants

**DOI:** 10.33549/physiolres.935622

**Published:** 2026-02-01

**Authors:** Endre SULYOK, Péter MAUCHART, Bálint FARKAS, Ákos VÁRNAGY, József BÓDIS

**Affiliations:** 1 National Laboratory on Human Reproduction, University of Pécs, Pécs, Hungary; 2 Faculty of Health Sciences, Doctoral School of Health Sciences, University of Pécs, Pécs, Hungary; 3 Department of Obstetrics and Gynecology, Medical School, University of Pécs, Pécs, Hungary

**Keywords:** Renal immaturity, Na^+^/H^+^ exchange, Hormonal regulation

## Abstract

Early clinical studies on renal acidifying processes and sodium reabsorption in premature infants were revisited in the context of Na^+^/H^+^ exchangers (NHEs) and their developmental regulation. Relevant literature was analyzed to highlight the physiological and clinical significance of NHEs in controlling Na^+^/H^+^ exchange during kidney maturation. The immature kidney’s low NHE capacity may lead to transient H^+^ retention, metabolic acidosis, sodium depletion, and late hyponatremia in premature infants. Sodium depletion excessively activates the renin-angiotensin-aldosterone system (RAAS), which stimulates NHE, reduces sodium loss, and mitigates late metabolic acidosis. Interestingly, NaCl supplementation suppresses systemic RAS activity while also preventing late metabolic acidosis, potentially through renal RAS-mediated NHE upregulation. Circulating and renal RAS may have distinct roles in regulating NHE activation and in maturity-related changes in sodium and acid-base homeostasis in premature infants.

## Introduction

The basic observations by Pitts and his co-workers that the renal tubular H^+^ secretion is associated with tubular Na^+^ reabsorption have established the key role of Na^+^-H^+^ exchange in the renal acidifying processes [1,2,3]. Since their discovery great advances have been made in our understanding of the underlying molecular mechanisms, regulation, ontogeny, pathophysiological, and clinical significance of the renal tubular Na^+^-H^+^ exchange. In light of the enormous new knowledge that accumulated during the last decades it appears to be justified to reconsider the results and to complete the interpretation of the unappreciated pioneer works by the group of E. Kerpel-Fronius on the postnatal development of renal acidifying processes in premature infants [4,5,6,6a].

## Some milestones of Na^+^/H^+^ antiporter research

Early studies have shown an electroneutral exchange of Na^+^ for H^+^ in the cristae membranes of mammalian mitochondria and a similar transport system has been detected in several bacterial strains [7,8,9].

Further studies by using micropuncture and renal brush-border membrane vesicles provided direct evidence for the function of Na^+^/H^+^ antiporter in the apical membrane of the renal proximal tubules [10,11].

An essential step of the NHE research was the identification of the NHE 1–9 isoforms, the genes encoding these proteins (SLC9A1–9) and exploring their tissue expression and cellular distribution. NHE1 was the first mammalian NHE isoform that has been widely studied. Its primary structure has been determined and its role to control intracellular pH, sodium level and cell volume, as well as its involvement in the regulation of cell cycle, proliferation, migration, adhesion and cell survival has been established [12,13,14].

The tissue-specific and time-dependent mRNA and protein expression of NHE1 has been documented in various tissues of fetal and neonatal mice including the heart, liver, lung, kidney and brain [15].

In addition to the kidney, important studies have been conducted to explore the role of NHE isoforms in mediating the electroneutral exchange of extracellular Na^+^ for intracellular H^+^ in *placental tissue*. In fact, NHE1, NHE2, and NHE3 have been detected in microvillous membrane of syncytiotrophoblast [16]. Their activity increases over gestation and the amiloride-sensitive Na^+^ uptake by the microvillous membrane is markedly elevated in term placenta as compared to the first trimester placenta [17]. Interestingly, when placental NHE1 activity and expression were compared between normally grown and growth-retarded preterm and full-term infants, both the expression and activity of NHE1 were lower in the growth-retarded group delivered preterm. The limited Na^+^/H^+^ exchange has been claimed to contribute to fetal acidosis frequently seen in these infants without apparent birth asphyxia [18].

The critical role of Na^+^/H^+^ exchanger isoforms in *brain* development has also been demonstrated. Their expression proved to be region-, age- and subtype- specific with varying levels of NHE1, NHE2, NHE3 and NHE4 in the rat central nervous system [19]. NHE1 isoform is the most prevalent and its excessive activation by hypoxia-ischemia results in intracellular Na overload, Ca^2+^ entry into the cells and activation of neurotoxic cascade. Furthermore, rapid normalization or even alkaline shift in intracellular pH induces enhanced excitability, cell death with cerebral atrophy and impaired outcome. The clinical relevance of these observations is underlined by experimental studies showing that neuroprotection can be achieved by NHE inhibition [20]. The possible involvement of NHE3 gene polymorphisms and its overexpression in the medulla oblongata may lead to altered breathing control and subsequently to sudden infant death syndrome [21,22].

Developmental regulation of *cardiac* NHE1 has been established in animal experiments. Accordingly, higher levels of mRNA for NHE1 were observed in the myocardium at fetal and neonatal stages than in adults, and similar developmental pattern was found for NHE1 activity in sarcolemmal vesicles obtained from rabbit hearts. Ballon injury to the ventricular wall and pressure overload on the ventricles increased NHE1 mRNA levels suggesting the involvement of NHE1 activation in cardiac hypertrophy and vascular smooth muscle cell proliferation [23]. Similar obser-vations were made in rats during postnatal development (2–42 days); there was a progressive decline in NHE1 mRNA expression with a concomitant reduction of sarcolemmal NHE1 activity during this age period [24]. Overexpression of NHE1 in the myocardium after hypoxia-reoxygenation challenge caused apoptosis [25] but inhibition of NHE attenuated the functional, morphological and biochemical derangement of pacing-induced heart failure in rabbits [23,26].

Studies on the *intestinal* distribution of human mRNAs for NHE1, NHE2 and NHE3 isoforms have revealed that the NHE1 message was uniformly present throughout the length of intestinal tract, whereas mRNAs for NHE2 and NHE3 isoforms demonstrated significant regional differences. Namely, the NHE3 abundance was the greatest in the ileum and declined steadily in the jejunum and the proximal and distal colon. NHE2 abundance was evenly distributed in the small intestine, but it was higher in the distal than in proximal colon. This tissue-specific localization of NHE2 and NHE3 mRNAs suggests that the relative contribution of these two isoforms in Na absorption may be region-specific and differentially regulated [27].

Genetic disruption of NHE3 expression in mice resulted in reduced intestinal Na absorption, mild metabolic acidosis, diarrhea and some fall in blood pressure [28]. Furthermore, intestinal NHEs modulate nutrient absorption and regulate microbial microen-vironment. When pathologies disrupt their function the dysregulated NHEs may contribute to disease progression [29]. Interestingly, dietary sodium depletion enhanced both NHE2 and NHE3 activity, protein and mRNA expression in proximal colon, while opposite effects were seen in the distal colon. NHE1 activity, protein and mRNA expression remained unaffected by sodium depletion. The differential effects of sodium depletion are tissue- and isoform-specific and appear to operate at pre-translational level [30].

Within the *kidney* five distinct NHE isoforms have been described: NHE1 – NHE4 and NHE8. They differ in their localization at various nephron segments, in their transport function and regulation.

NHE3 has been documented as the predominant Na^+^/H^+^ exchanger isoform of proximal tubular renal brush border vesicles accounting for more than 50 % of renal tubular sodium reabsorption. The molecular structure, functional characteristics, transcriptional/post- transcriptional regulation, and clinical significance of NHEs are extensively presented in basic articles and comprehensive reviews [31–39].

Concerning early studies on the postnatal development of Na^+^/H^+^ exchange in premature infants that were performed long before the discovery of NHEs, it has been clearly defined that the capacity of immature kidneys to reabsorb bicarbonate is markedly reduced and that proximal tubular bicarbonate reabsorption is largely dependent on sodium-dependent apical proton secretion. As these processes are predominantly mediated by the NHE3 antiporter a series of important animal studies has been conducted to reveal its developmental pattern and hormonal regulation [40,41].

As shown by the parallel increase of apical NHE3 activity, NHE3 mRNA expression, NHE3 protein levels and the subsequent improvement of sodium reabsorption, the postnatal maturation of NHE may be accelerated by endogenous and/or exogenous adrenocortical steroid [42,43,44,45], thyroid hormone [46], catecholamine [47,48] and angiotensin II stimulation [49,50,51]. In a series of recent studies Li *et al.* by using mouse model of specific deletion of proximal tubular NHE3 provided firm evidences for its important role in blood pressure regulation and angiotensin II-induced hypertension [52,53,54,55].

By contrast, dopamine that is frequently used in neonatal intensive care, induces natriuresis by inhibiting NHE3 activity in proximal tubular brush-border membrane vesicles *via* adenylate cyclase activation [56]. Moreover, tonic inhibition of renal tubular NHE3 by dopamine may be operating either directly *via* its own receptors or indirectly by interaction with other hormones (Table 1) [57,58].

NHE8 has also been shown to be expressed in the apical membrane of the proximal tubule and its protein abundance peaked at 7-and 14-day-old compared with adult rats. It was suggested, therefore, that NHE8 may have a role in Na-dependent proton flux in neonatal proximal tubule [59]. This contention has been confirmed by demonstrating that in response to metabolic acidosis neonatal NHE3-null mice had an increase in NHE8 protein abundance and NHE8 activity in brush-border membrane vesicles. Similar response pattern of NHE3 protein expression and activity could be seen in neonatal NHE8-null mice [60]. On the basis of these observations it can be assumed that this mutual compensatory increase of NHE3 and NHE8 in neonatal proximal tubules may serve as one of the underlying mechanisms of the metabolic acidosis-induced acceleration of renal acidifying processes in developing kidney [60]. It is to be noted that Joseph *et al.* have reported that glucocorticoids, known to enhance NHE3 expression and activity, reduced NHE8 expression in the neonates providing evidence for the inverse regulation of these two renal NHE isoforms by glucocorticoids [61].

## Comments on the clinical relevance of Na^+^/H^+^ ontogeny

1) In the early seventies it has been demonstrated that healthy premature infants have low renal capacity to secrete H^+^ and to reabsorb Na^+^. During the first weeks of postnatal life, these two renal functions have shown opposite trends and time courses suggesting progressive improvement of renal tubular Na^+^*/*H^+^ exchange. As a result, transient H^+^ retention and late metabolic acidosis developed accompanied by sodium depletion and late hyponatraemia [4,5]. A similar response pattern was seen after NH_4_Cl-induced metabolic acidosis but of course at significantly higher levels. It is of note that the Na^+^/H^+^ ratio has never been 1 to 1 implying that the renal excretion of sodium and/or hydrogen ions has also to be controlled by mechanisms other than Na^+^/H^+^ exchange in the proximal tubule. Concerning the involvement of corticosteroids in the maturation of NHE3 exchanger isoform convincing evidence has been provided that in animal models prenatal administration or postnatal surge of corticosteroids accelerates this process [42,43,44,45]. With these observations in line later studies on the postnatal course of plasma levels of adrenocortical steroids in premature infants revealed a slightly higher cortisol, cortisone, and corticosterone levels in the first week than in the next four weeks [62].

In an attempt to reveal the involvement of glucocorticoids in the control of renal tubular sodium transport during development it has been demonstrated that the cyclic mineralocorticoid receptor (MR) expression tightly correlated with the evolution of 11-β-hydroxysteroid dehydrogenase 2 (11-β-HSD2) and α-epithelial sodium channel (α-ENaC) implying that the low renal MR expression in the perinatal period may limit renal tubular responsiveness to aldosterone and may account for the compromised sodium handling by the immature kidney [63,64].

The contribution of the renal sympathetic nervous system appears also to be plausible as urinary excretion of noradrenaline is significantly elevated when the Na^+^/H^+^ exchange undergoes progressive maturation [65].

2) To reveal the underlying mechanisms of the maturation pattern of renal sodium handling the proximal and distal tubular sodium reabsorption was assessed separately by using clinically relevant clearance methods. It has been found that in full term neonates both the proximal and distal tubular sodium reabsorption proved to be significantly more efficient than in premature [66]. Furthermore, in premature infants distal tubular sodium delivery (as a measure of proximal reabsorption) steadily decreased during the study period of 6 weeks, whereas distal sodium reabsorption abruptly rose by the 2^nd^ week and remained practically unchanged thereafter [67]. The forced stimulation for distal tubular sodium reabsorption was claimed to be attributed to the excessively activated RAAS [68,69,70].

During the following years much effort has been made to explore the developmental regulation of the distal tubular sodium reabsorption at cellular levels. The inefficient active sodium transport by the immature distal nephron has been assumed to be due to intracellular mechanisms including incomplete polarization of the principal cells, decreased number and conductance of epithelial sodium channels (ENaCs), and low activity of basolateral Na^+^, K^+^-ATPase [71,72,73,74]. In this regard the longitudinal, prospective study by Delgado *et al.* is of particular interest. In premature infants with the gestational age of 23 to 31 weeks they could document clinical-molecular correlates of developmental regulation of renal sodium handling. Namely, the progressive fall in fractional sodium excretion with gestational and postnatal ages was found to be associated with a significant increase of about 25 % in α-ENaC mRNA abundance [75], the transcript of major subunit of channel protein, α-ENaC that mediates distal tubular sodium reabsorption [76].

3) Attempts have also been made to explore the involvement of RAAS in control of sodium balance during the neonatal period. The renal salt wasting and the subsequent sodium depletion resulted in excessive activation of the renin-angiotensin aldosterone system (RAAS) to mitigate further sodium loss and to re-establish normal sodium balance. It has been thought to be achieved by forced stimulation of sodium reabsorption in the aldosterone-responsive distal tubule. Accordingly, the urinary sodium/potassium ratio, a clinical marker of renal tubular responsiveness to aldosterone, is greatly elevated in the first week followed by a progressive decline in the subsequent weeks in these healthy preterm neonates [68]. Further evidence for the limited response to aldosterone stimulation has been provided in one-week-old neonates with gestational age of 30–41 weeks by showing that urinary aldosterone excretion is negatively related to urinary Na excretion and urinary Na^+^/K^+^ ratio. In this particular group of human neonates it was found that PRA declined progressively with advancing gestation (p<0.001) and it positively related to urinary sodium loss (r=0.71, p<0.001) and inversely to sodium balance (r=−0.57, p<0.001) providing strong evidence for the adequate PRA response to the sodium depleted state [69]. With good agreement with these observations, Miyawaki *et al.* described essentially the same developmental pattern for plasma AngII concentration in human neonates on day 7 of life. Specifically, apparent negative correlations have been observed between AngII concentration and gestational age (r=−0.4, p<0.007) and the respective birth weight (r=−0.36, p<0.016) [70].

4) The relationship between the postnatal development of RAAS, and the electrolyte and acid-base balance was further explored in premature infants supplemented with NaCl. In our clinical practice supplementation was initiated on the 7^th^ day and continued until the 6^th^ week of life. Depending on the birthweight of preterm neonates it was given in a dose of 3–5 mEq/kg/day and 1.5–2.5 mEq/kg/day for 8–21 days and 22–35 days, respectively, aiming to compensate for renal Na^+^ loss and to prevent sodium depletion and hyponatraemia. In response to NaCl supplementation positive Na^+^ balance and normonatraemia could be maintained, but the activity of RAAS was markedly depressed. Unexpectedly, after NaCl supplementation late metabolic acidosis did not develop as indicated by the significantly higher plasma total CO_2_ levels and lower base deficit when compared with those of non-supplemented premature infants. These latter findings strongly suggest that in these clinical settings, NaCl supplementation enhances renal tubular Na^+^-H^+^ exchanges [6].

In light of the significant reduction in circulating RAAS elements in premature infants on high sodium intake, the increased activity of the proximal tubular Na/H exchanger appears to be paradoxical as angio-tensin II is one of the major factors stimulating this renal transporter. Instead, one can assume that the increased tubular sodium load directly stimulates NHE activity independent of angiotensin II. This assumption is consistent with the notion that Na^+^-specific environment-tal signals may turn on NHE [35]. Accordingly, in NaCl supplemented premature infants extracellular Na^+^ may prevail over intracellular H^+^ in stimulating Na^+^ reabsorption and H^+^ secretion (Table 2).

An alternative/additional mechanism to achieve effective Na^+^/H^+^ exchange may be encountered by the local renin-angiotensin system (RAS) that is present in several organs/tissues and acts in an autocrine/paracrine fashion. *Via* the classical pathway, angiotensin II (AngII) stimulates AT1R and induces vasoconstriction, sodium retention, reactive oxygen species generation, inflammation, and fibrosis. By contrast, the ACE2-Ang(1–7)-MasR axis of the alternative pathway has opposing effects and serves as a protective arm of RAS. The actions of the two arms of RAS are well-balanced and tightly regulated. Unopposed stimulation of AT1R may be deleterious with untoward clinical consequences.

The effects of dietary sodium on renal RAS have been extensively studied with conflicting results. Renal renin and angiotensinogen mRNA expressions were significantly elevated in sodium depleted vs high sodium fed rats [77,78,79,80]. Importantly, irrespective of the down regulation of renal renin mRNA expression the transcripts of AT1R were more abundant in rats receiving high salt diet as compared with those on low salt intake [81]. It is of particular interest that high sodium intake increased renal AngII levels and reduced the expression of the elements of ACE2-Ang(1–7)-MasR axis favoring unopposed sodium reabsorption in obese Zucker rats [82].

**Fig. 1:**
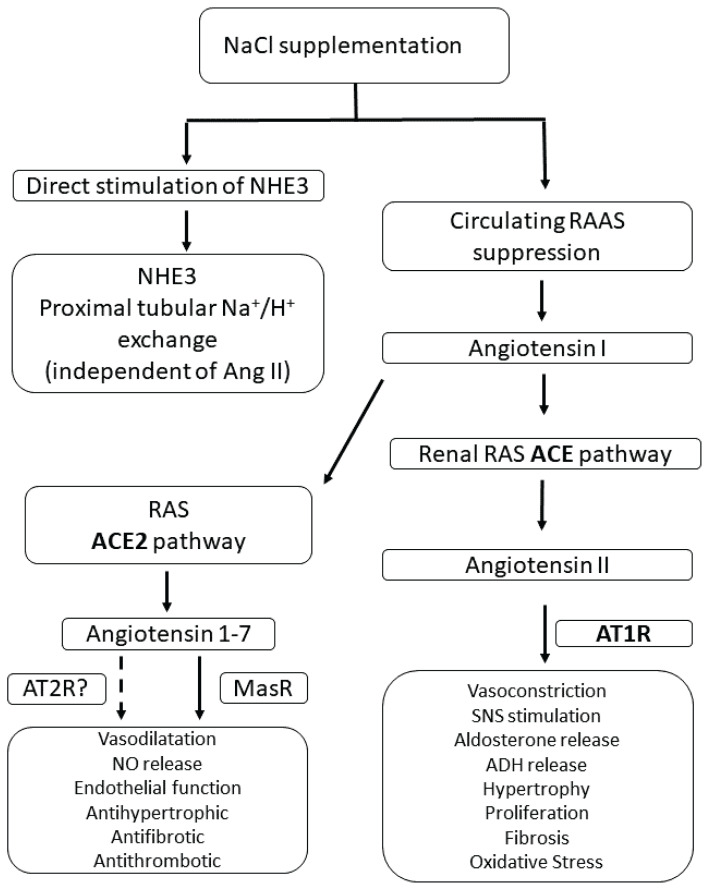
The effects of NaCl supplementation on the renal renin-angiotensin system (RAS) and NHE3 activity. NaCl supplementation exerts two major effects: (1) direct stimulation of NHE3 in the proximal tubule, independent of angiotensin II (AngII), enhancing Na^+^/H^+^ exchange; and (2) suppression of the RAS ACE pathway, reducing AngII levels. AngII acts through the AT1R receptor, promoting vasoconstriction, fibrosis, oxidative stress, and other detrimental effects. In parallel, the ACE2 pathway converts AngII into Angiotensin-(1–7), which interacts with the Mas receptor (MasR) and possibly with the AT2 receptor (AT2R) to induce vasodilation, nitric oxide release, improved endothelial function, antihypertrophic, antifibrotic, and antithrombotic effects. This balance highlights the opposing roles of the classical RAS and the protective RAS.

Recent human studies have shown that neonates and adolescents born preterm have lower plasma and/or plasma and urinary Ang(1–7) levels and a higher AngII to Ang(1–7) ratio when compared with those born at term. It has been suggested, therefore, that the predominance of the classical RAS pathway over the protective ACE2-Ang (1–7)-MasR pathway may result in enhanced AngII-AT1R receptor mediated proximal tubular Na^+^/H^+^ exchange in spite of markedly suppressed circulating RAAS. Na supplementation in premature infants may be a further factor inducing a shift from protective to classical renal RAS [83] (Fig. 1). AT2 receptor is maximally expressed at about 8 weeks of gestation followed by decreasing but persistent expression until about 20 weeks of gestation, therefore the activation of AT2R pathway might play some role in regulation of NHE in early life of premature infants [84].

## Conclusions

The sodium and acid-base homeostasis is interdependent and tightly controlled by common mechanisms. Ample evidences indicate the principal role of NHE3 located in the apical membrane of the renal proximal tubular cells. It mediates H^+^ secretion in exchange for Na^+^ under the control of several hormones. Experimental studies have shown that the mRNA and protein expressions and also the activity of NHE3 are markedly depressed in the immature kidney and steadily increase with age. These observations confirm early clinical studies demonstrating the developmental pattern of Na^+^-H^+^ exchange and its relation to late metabolic acidosis and late hyponatraemia in premature infants. The progress-sive increase of Na^+^-H^+^ exchange was thought to be due to the excessively activated RAAS. Unexpectedly, NaCl supplementation had similar effects despite the reduced activity of circulating RAAS. This paradoxical reaction may be accounted for by direct stimulation of NHE by sodium load and/or by the shift of protective (ACE2-Ang(1–7)-MasR) to the classical renal RAS (ACE-AngII-AT1R) (Table 3).

## Figures and Tables

**Table 1:** Hormonal effects on NHE3 activity.

*Hormone*	Effect on NHE3 Activity	Mechanism
*Glucocorticoids*	Stimulatory	Increases NHE3 activity in neonatal kidneys (*via* stimulation of NHE3 mRNA and protein expression)
*Angiotensin II*	Stimulatory	Activates AT1 receptor
*Thyroid hormone*	Stimulatory	Increases NHE3 activity in neonatal kidneys (*via* stimulation of NHE3 mRNA and protein expression)
*Dopamine*	Inhibitory	Activates adenylate cyclase

**Table 2:** NaCl supplementation effects.

*Group*	Plasma Sodium Level	RAAS Activity	Metabolic Acidosis
*NaCl supplemented*	Normal	Decreased	Less pronounced
*No supplement*	Reduced	Increased	Unchanged

**Table 3:** A summary of the key points and conclusions.

*Topic*	Conclusion	Location
*Role of NHE3*	Mediates Na^+^/H^+^ exchange, critical for acid-base balance and sodium reabsorption.	Proximal tubule, apical membrane (NHE3)
*Role of NHE8*	Compensates for the reduced NHE3 activity during early kidney development.	Proximal tubule, apical membrane (NHE8)
*Ontogenesis*	NHE3 activity increases with age, under the regulation of several hormones	Proximal tubule (NHE3, NHE8)
*Effect of NaCl supplementation*	Paradoxically enhances NHE3 activity even with reduced circulatory RAAS, increasing sodium reabsorption.	Proximal tubule (NHE3)
*Clinical relevance*	Limited bicarbonate reabsorption in immature proximal tubules contributes to metabolic acidosis, while low ENaC expression in distal tubules limits sodium reabsorption.	Proximal and distal tubules
